# Thirty-two-year trends of cancer incidence by sex and cancer site in the Veneto Region from 1987 to 2019

**DOI:** 10.3389/fpubh.2023.1267534

**Published:** 2024-01-08

**Authors:** Alessandra Buja, Giuseppe De Luca, Manuel Zorzi, Emanuela Bovo, Simone Mocellin, Chiara Trevisiol, Vincenzo Bronte, Stefano Guzzinati, Massimo Rugge

**Affiliations:** ^1^Department of Cardiological, Thoracic, Vascular Sciences and Public Health, University of Padua, Padua, Italy; ^2^Veneto Tumour Registry, Azienda Zero, Padua, Italy; ^3^Soft-Tissue, Peritoneum, and Melanoma Surgical Oncology Unit, Veneto Institute of Oncology IOV – IRCCS, Padua, Italy; ^4^Department of Surgery, Oncology, and Gastroenterology – DISCOG, University of Padova, Padua, Italy; ^5^Veneto Institute of Oncology IOV – IRCCS, Padua, Italy; ^6^Pathology and Cytopathology Unit, Department of Medicine-DIMED, University of Padua, Padua, Italy

**Keywords:** epidemiology, cancer, sex characteristics, observational study, cohort studies, incidence, registry

## Abstract

**Background:**

This observational study considers the sex-specific incidence of the most incident cancers as recorded in the population-based Veneto Regional Cancer Registry over a period of more than 30 years (1987-2019).

**Methods:**

The Veneto Regional Cancer Registry collected data for the time interval 1987–2019. Significant changes in incidence trends calculated on age-standardized incidence rates (Annual Percent Change—APC) were identified by join point regression analysis.

**Results:**

Overall, the incidence trend for all cancers decreased in males and remained stable in females. In nine cancer sites, the incidence trends showed consistent differences by sex (oral cavity, esophagus, colon rectum and anus, liver, larynx, lung, cutaneous malignant melanoma, bladder, and thyroid gland). Other malignancies did not show significant sex-related differences (stomach, pancreas, biliary tract, kidney/urinary tract, central nervous system, multiple myeloma, non-Hodgkin lymphoma, and leukemia).

**Conclusion:**

In the period 1987–2019, this study revealed sex-related differences in cancer incidence trends. Over time, cancer incidence remained higher in males, with a decreasing epidemiological impact, plausibly resulting from prevention campaigns against environmental cancer risk factors, as tobacco and alcohol. Conversely, a significant decrease was not observed in the incidence trend in females. These findings contribute essential insights for profiling the epidemiological map of cancer in a large Italian population, allowing comparison with other European cancer epidemiology studies and providing updated data supporting sex-related primary and secondary cancer prevention strategies.

## 1 Introduction

Globally, 18,094,716 million cancer cases were diagnosed in 2020. Among these, the incidence rate for all cancers combined was 19% higher in males (222.0 per 100,000) than in females (186.0 per 100,000) (1). Previous studies have also reported higher cancer incidence rates in males at nearly all ages and in most countries (2–4). In Italy, the combined incidence rate was 16% higher in males (317.5 per 100,000) than in females (274.8 per 100,000) (1). The available Italian data on cancer incidence trends by sex support a significant decrease in males and a stable trend in females (5).

Sex and age play an important role in the epidemiology of cancer but they are not always considered in the cancer management. Few recent, large-scale estimates specifically focus on cancer incidence trends by sex across all cancer site. Many of them use less comprehensive dataset due to the limited number of tumor site (6) or time horizon (7–9) or are limited to a specific age (i.e. childhood, young adults and older adults) (10–12). Systematic assessment of sex-specific incidence trends over a large time horizon can provide important information about sex-specific cancer epidemiology, its temporal variability, and the priorities for gender-tailored preventive interventions.

In the north east of Italy, the Veneto region covers approximately 18,345 km^2^, with a resident population of over 4.8 million. Mortality, measured by the standardized death rate, is lower than the national average (7.9 vs. 8.2 per 1,000 inhabitants in 2016) (13). The main causes of death are cancer and cardiovascular disease (14).

The Regional healthcare system is based on the fundamental values of universality, free access, freedom of choice, pluralism of supply, and equity (14).

This population-based cohort study focused on sex-related differences in the incidence trends of malignancy by site, during a time span of just over 30 years (1987–2019), in a large northern Italian population.

## 2 Materials and methods

### 2.1 Data sources

This retrospective population-based cohort study draws on epidemiological data of cancer incidence recorded by the Veneto Cancer Registry (RTV-*Registro Tumori del Veneto*) from 1987 to 2019. The RTV was certified by the International Agency for Research on Cancer with excellent quality indicators. In the last publication the percentage of cases microscopically verified was greater than 88% and the percentage of cases with death certificate only was less than 1% (15). Population coverage increased from 1,154,000 inhabitants in 1987 to nearly five million (encompassing the entire regional population) since 2014 (see Supplementary Table S1).

Cancer registration methods have also been updated during the period from the International Classification of Diseases, 9th Revision (ICD-9) to ICD-10, and from ICD-O-2 to ICD-O-3 (16). The temporal trends in cancer incidence (standardized for the European population in 2013) were calculated based on the tumor and sex of the patient.

RTV records all malignancies—considering only invasive cutaneous malignant melanoma (CMM) among the skin malignancies—and non-malignant tumors of the urinary bladder, identified according to ICD-10 (17).

Official population data are made available from Italian National Institute of Statistics (18).

### 2.2 Statistics

A joinpoint regression analysis was performed to identify any significant changes in the annual trends (using the 2013 European Standard Population) of standardized incidence rates (ASR) for the most frequent cancer sites, stratified by sex (19). For each identified trend, the annual percent change (APC) was calculated by fitting a regression line to the natural logarithm of the rates, using the calendar year as a regression variable. The graph for each tumor model depicts statistically significant changes in trend.

To verify the cancer incidence homogeneity among different areas covered form the Registry in the whole period analyzed we calculated the rate ratio (RR), i.e., the ratio between the ASR of the Region and the ASR of the historical area by sex and sites in the last 5 years analyzed. Historical area (HA) is defined as the population covered by the Registry since 1990, representing 45% of the whole regional population. RR was also used to test differences in ASR between sexes.

The significance level is set to 0.05 for all tests.

The statistical analysis was performed using Joinpoint Regression Program, 4.6.0 version (2018) and SEER*Stat, 8.4.2 version (2023).

## 3 Results

### 3.1 Current epidemiological profile

In 2019, the RTV recorded 31,925 incident malignancies [males (M) = 16,725; females (F) = 15,200; Table 1]. In males, the five most frequent cancer sites (prostate, lung/bronchus/trachea, colon/rectum/anus, urinary bladder, and CMM) accounted for 59.5% of all malignancies. In females, breast malignancies accounted for 34% of all incident cancers, followed by the colorectum, lung, corpus uteri, and thyroid (overall frequency = 60%).

**Table 1:** Cancer incidence in the year 2019, by tumor site and sex in Veneto Region, Italy.

	Male		Female	
Tumor site (ICD-10)	N. of cases	ASR^1^	N. of cases	ASR^1^
Oral cavity (C00–C14)	474	18.4	255	8.2
Esophagus (C15)	187	7.3	72	2.2
Stomach (C16)	495	19.5	355	10.8
Colon, rectum and anus (C18–C21)	1,829	71.7	1,517	46.1
Liver and intrahepatic bile ducts (C22)	647	25.3	252	7.9
Pancreas (C25)	603	23.6	578	16.9
Larynx (C32)	254	9.8	35	1.2
Lung, bronchus and trachea (C33, C34)	1,879	74.6	1,072	34.3
Invasive cutaneous melanoma (C43)	883	34.3	662	23.7
Breast (C50)			5,173	175.4
Corpus uteri (C54)			723	24.5
Prostate (C61)	3,746	146.3		
Central nervous system (C70–C72)	287	11.2	215	7.4
Thyroid gland (C73)	291	11.4	692	26.7
Kidney and urinary tract (C64–C66, C68)	805	31	423	13.9
Bladder (C67, D09.0, D41.4)	1,623	63.8	463	14.4
Non-Hodgkin lymphoma (C82–C86, C96)	602	23.6	498	16.6
Multiple myeloma and immunoproliferative diseases (C88–C90)	313	12.2	267	8.5
Leukemia (C91–C95)	319	12.8	231	7.6
All malignant tumors excluding *in situ* cutaneous melanoma and non-melanoma skin cancer (C44)	16,725	656.9	15,200	502.6

^1^Age-standardized rate (European standard population 2013).

Age-standardized incidence rates were significantly higher in males, except for thyroid cancer (*p* < 0.001).

### 3.2 Temporal trends in incidence

From 1987 to 2019, the regional population ranged from 4,498,402 to 4,906,000. From its establishment in 1987 until 2013, cancer registration included about half of the population, reaching full regional population coverage from 2014 onwards (see Supplementary Table S1). Supplementary Table S2 shows that the historical registration area yielded incidence estimates for different cancer sites which have proven comparable with those available for the whole population. Only for the Urinary Bladder in women was a significant difference found (*p* = 0.04). The standardized rate of all incident malignancies in the interval times of 1987–2013 and 2013–2019 was 690.0 vs. 690.7 for males, and 516.5 vs. 512.8 for females, with rate ratios of 1.001 and 0.9929, respectively (both *p* > 0.05).

**Figure 1:**
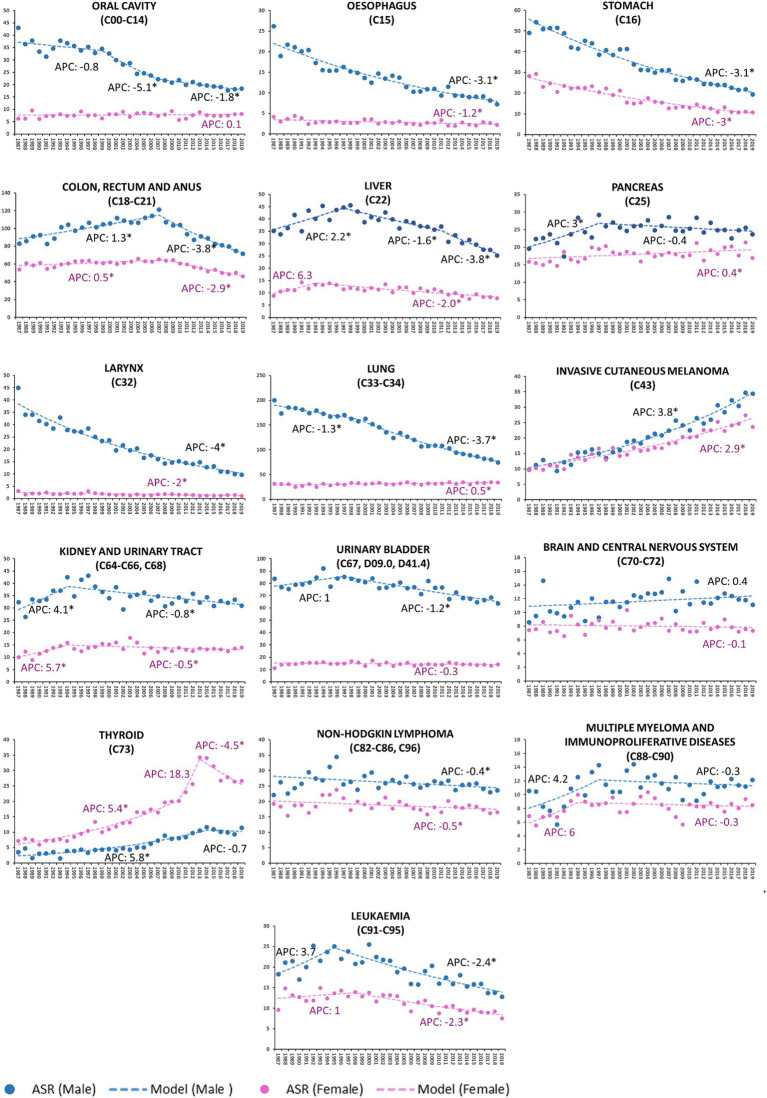
Age-standardized incidence rate (ASR) per 100,000 residents (2013 European standard population) and annual percent change (APC) by tumor site and by sex in Veneto Region, Italy from 1987 to 2019. Cancers are listed according to ICD-10 classification. *Statistically significant value (*p* < 0.05).

**Figure 2:**
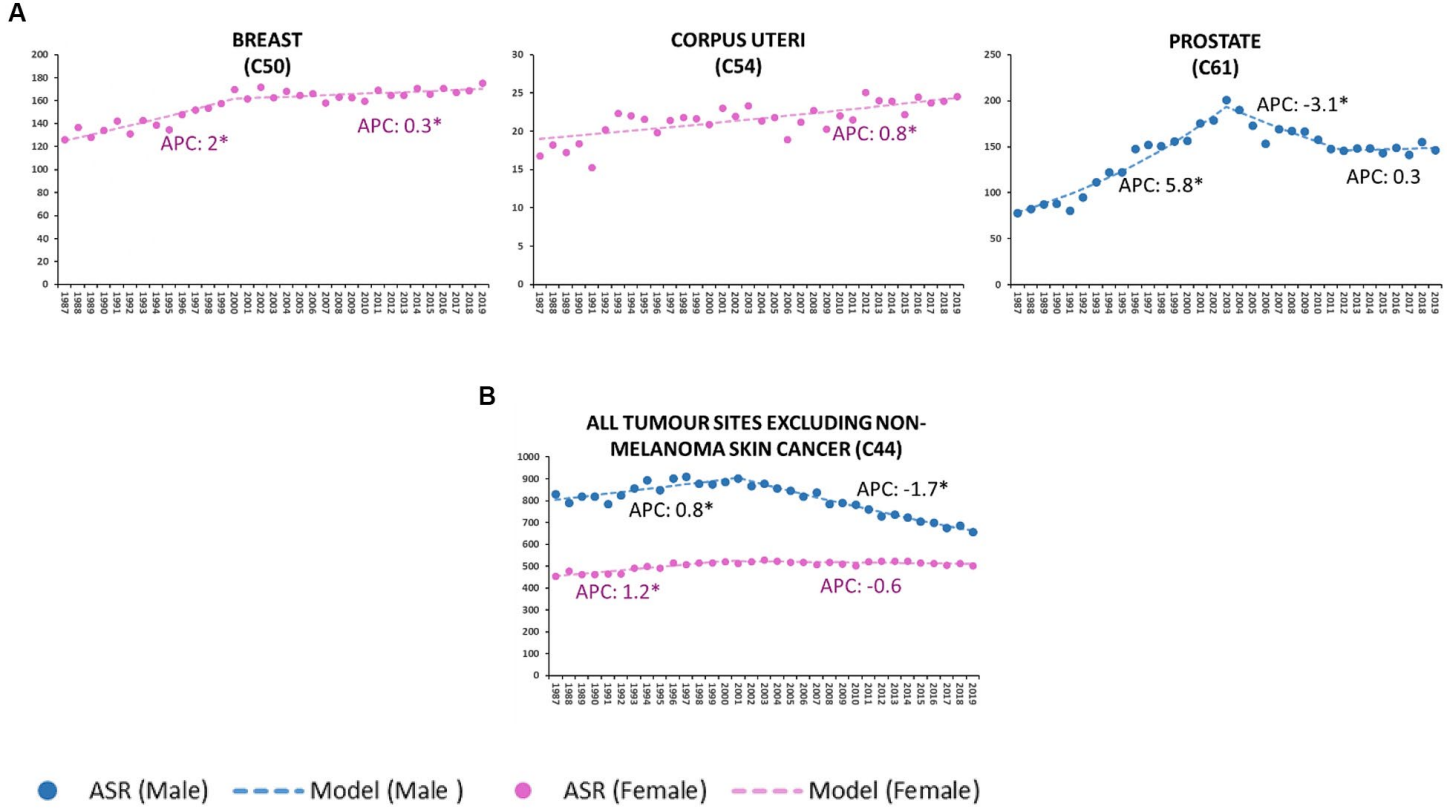
Age-standardized incidence rate (ASR) per 100,000 residents (2013 European standard population) and annual percent change (APC) of breast, corpus uteri, prostate sites **(A)**, all malignant tumors except in situ cutaneous melanoma and non-melanoma skin cancer (C44) **(B)**. Veneto Region, Italy from 1987 to 2019. *Statistically significant value (*p* < 0.05).

During the considered time interval, the cancer incidence trends by site showed significant sex-related differences both in not sex-specific malignancies (Figure 1; Table 2) and sex-linked hormone-dependent cancers (Figure 2A; Table 2).

**Table 2:** Joinpoint regression models of individual cancer diagnoses by tumor site and by sex in Veneto Region, Italy

	Male			Female		
Tumor site (ICD-10)	Period	APC	(95 % CI)	Period	APC	(95 % CI)
Oral cavity (C00–C14)	1987–1999	−0.8	(−1.7; 0.2)	1987–2019	0.1	(−0.3; 0.5)
	1999–2007	−5.1*	(−7.0; −3.2)			
	2007–2019	−1.8*	(−2.5; −1.0)			
Esophagus (C15)	1987–2019	−3.1*	(−3.4; −2.9)	1987–2019	−1.2*	(−1.7; −0.6)
Stomach (C16)	1987–2019	−3.1*	(−3.3; −2.9)	1987–2019	−3.0*	(−3.3; −2.7)
Colon, rectum and anus (C18–C21)	1987–2007	1.3*	(1; 1.7)	1987–2008	0.5*	(0.2; 0.8)
	2007–2019	−3.8*	(−4.3; −3.4)	2008–2019	−2.9*	(−3.5; −2.4)
Liver and intrahepatic bile ducts (C22)	1987–1997	2.2*	(0.6; 3.9)	1987–1993	6.3	(−0.6; 13.6)
	1997–2011	−1.6*	(−2.4; −0.9)	1993–2019	−2.0*	(−2.4; −1.5)
	2011–2019	−3.8*	(−5.1; −2.5)			
Pancreas (C25)	1987–1997	3.0*	(0.4; 5.5)	1987−2019	0.4*	(0.1; 0.8)
	1997–2019	−0.4	(−0.8; 0)			
Larynx (C32)	1987–2019	−4*	(−4.3; −3.8)	1987–2019	−2.0*	(−2.4; −1.3)
Lung, bronchus and trachea (C33, C34)	1987–1999	−1.3*	(−2; −0.7)	1987–2019	0.5*	(0.3; 0.7)
	1999–2019	−3.7*	(−3.9; −3.5)			
Invasive cutaneous melanoma (C43)	1987–2019	3.8*	(3.5; 4.1)	1987−2019	2.9*	(2.6; 3.2)
Prostate (C61)	1987–2003	5.8*	(4.8; 6.8)	–	–	–
	2003–2012	−3.1*	(−4.6; −1.6)	–	–	–
	2012–2019	0.3	(−1; 1.6)	–	–	–
Breast (C50)	–	–	–	1987–2000	2.0*	(1.4; 2.5)
	–	–	–	2000–2019	0.3*	(0.1; 0.5)
Corpus uteri (C54)	–	–	–	1987–2019	0.8*	(0.5; 1)
Ovary (C56)	–	–	–	1987–1996	6.6*	(3.3; 9.9)
	–	–	–	1996–2019	−1.2*	(−1.7; −0.7)
Kidney and urinary tract (C64–C66, C68)	1987–1994	4.1*	(0.1; 8.3)	1987–1994	5.7*	(0.4; 11.3)
	1994–2019	−0.8*	(−1.2; −0.5)	1994–2019	−0.5*	(−1; 0)
Urinary bladder (C67, D09.0, D41.4)	1987–1997	1	(−0.5; 2.5)	1987–2019	−0.3	(−0.5; 0)
	1997–2019	−1.2*	(−1.5; −1)			
Brain and central nervous system (C70–C72)	1987–2019	0.4	(0; 0.8)	1987–2019	−0.1	(−0.5; 0.2)
Thyroid gland (C73)	1987–2014	5.8*	(4.8; 6.9)	1987–2010	5.4*	(4.7; 6.3)
				2010–2013	18.3	(−2.9; 43.9)
	2014–2019	–0.7	(−6; 4.6)	2013–2019	−4.5*	(−6.6; −2.4)
Non–Hodgkin lymphoma (C82–C86, C96)	1987–2019	−0.4*	(−0.7; −0.1)	1987–2019	−0.5*	(−0.8; −0.1)
Multiple myeloma and immunoproliferative diseases (C88–C90)	1987–1997	4.2	(−0.2; 8.9)	1987−1994	6	(−0.6; 13.2)
	1997–2019	−0.3	(−1; 0.4)	1994–2019	−0.3	(−0.9; 0.3)
Leukemia (C91–C95)	1987–1995	3.7	(−0.4; 8)	1987–1998	1	(−1.1; 3.2)
	1995–2019	−2.4*	(−2.9; −1.9)	1998–2019	−2.3*	(−2.9; −1.8)
All malignant tumors excluding *in situ* cutaneous melanoma and non-melanoma skin cancer (C44)	1987–2001	0.8*	(0.5; 1.2)	1987–1997	1.2*	(0.8; 1.6)
	2001–2019	−1.7*	(−1.9; −1.6)	1997−2019	−0.6	(−1.3; 0.1)

APC, annual percent change; CI, confidence interval. *Statistically significant value (*p* < 0.05).

From 2001 onwards, the incidence of all-site malignancies in males declined steadily (M-APC: −1.7, 95%C.I.: −1.9, −1.6); since 1997, no significant changes in incidence have been recorded in females (Figure 2B).

The incidence of oral cavity cancer consistently decreased in males only (M-APC: −1.8, 95%C.I.: −2.5; −1.0 [2007–2019] vs. F-APC: 0.1, 95%C.I.: −0.3; 0.5 [1987–2019]).

From 1987 to 2019, the decline of APC in esophageal cancer was three times higher in males than females (M-APC: −3.1, 95%C.I.: −3.4; −2.9 vs. F-APC: −1.2, 95%C.I.: −1.7; −0.6).

Colorectal and anal cancers decreased more significantly in males (M-APC: −3.8, 95%C.I.: −4.3; −3.4 [2007–2019] vs. F-APC −2.9, 95%C.I. −3.5; −2.4 [2008–2019]).

The incidence of liver malignancies fell more significantly among males (M-APC: −3.8, 95%C.I.: −5.1; −2.5, [2011–2019] vs. F-APC −2.0, 95%C.I.: −2.4; −1.5 [1993–2019]).

Over the time interval 1987–2019, laryngeal cancers consistently displayed a decreasing incidence trend, and the decline of APC was twofold higher in males than in females (M-APC: −4.0, 95%C.I.: −4.3; −3.8 vs. F-APC −2.0, 95%C.I.: −2.4; −1.3).

Lung cancer decreased in males (M-APC: −3.7, 95%C.I.: −3.9; −3.5, [1999–2019]), while a slight, but significant increase was recorded in females (F-APC: 0.5, 95%C.I.: 0.3; 0.7 [1987–2019]).

During the reported time interval (1987–2019), invasive cutaneous melanoma showed a growing trend, particularly in males (M-APC: 3.8, 95%C.I.: 3.5; 4.1 vs. F-APC: 2.9, 95%C.I.: 2.6; 3.2).

Urinary bladder malignancies in males consistently showed a decreasing trend (M-APC: −1.2, 95%C.I.: −1.5; −1.0 [1997–2019]); since 1987, no significant modifications in incidence were recorded in females.

From 1987 to 2014, the incidence of thyroid cancer rose steadily in males (M-APC: 5.8, 95%C.I.: 4.8; 6.9) and subsequently stabilized. Incidence in females showed a two-phase increasing trend up to 2013 (APC: 5.4, 95%C.I.: 4.7; 6.3 [1987–2010]; APC: 18.3, 95%C.I.: −2.9; 43.9 [2010–2013]), followed by a steep decline (APC: −4.5, 95%C.I.: −6.6; −2.4 [2013–2019]).

Between 2000 and 2019, the incidence of breast cancer displayed a slight but steady increase (APC: 0.3, 95%C.I.: 0.1; 0.5), differing substantially from the sharp rise recorded in the period 1987–2000 (APC: 2.0, 95%C.I.: 1.4; 2.5).

Throughout the considered time interval (1987–2019), the incidence of corpus uteri malignancies consistently increased (APC: 0.8, 95%C.I.: 0.5; 1).

Prostate cancer rose significantly between 1987 and 2003 (APC: 5.8, 95%C.I.: 4.8, 6.8), followed by a downturn in the decade 2003–2012 (APC: −3.1, 95C.I.: −4.6, −1.6); no notable changes were observed in the last 10 years.

## 4 Discussion

This study explores cancer incidence trends by site and sex as recorded in the population-based Veneto Regional Cancer Registry from 1987 to 2019. Overall cancer incidence declined in both sexes, and the more significant decrease observed in men lowered the “historical” gap between males and females (20).

### 4.1 Divergent incidence trends by sex

As in other high-income countries (21–24), environmental cancers, particularly those related to tobacco exposure, showed a divergent trend of incidence by sex. The incidence of lung cancer decreased significantly in males, while marginally increasing in females. These features are consistent with the decline in tobacco consumption recorded among Italian males since 1970. During the same time interval, the percentage of female smokers steadily rose (25). Moreover, the decreasing incidence in males, as recorded by RTV since the late 1980s, is biologically consistent with the 20–30-year latency of tobacco-related cancer development reported in one other study (26).

Similar opposing sex-related trends were observed for cancers arising from the oral cavity and urinary bladder (27, 28), both sharing the same environmental risk factor(s) (29–31).

### 4.2 Decreasing incidence trends in both sexes

Many cancer sites showed consistently decreasing trends in both sexes, even at different magnitudes.

Globally, the incidence of thyroid cancer proved considerably higher among females. In 2020, the age-standardized incidence rates of thyroid malignancies were 10.1 and 3.1 per 100,000 among females and males, respectively (32). From 1987 to 2014, RTV recorded a steadily increasing incidence in males, followed by a slight decline. From 1987 to 2013, the incidence of female thyroid malignancies rose significantly, followed by a dramatic sixfold decrease compared to males. Similar findings were reported in other studies (33, 34), being critically interpreted to stem from newly established (more stringent) diagnostic criteria rather than from changes in clinical-biological risk factors (35–39). The proportion of thyroid cancer due to overdiagnosis between 2008 and 2012 was in fact estimated to be higher than 70% in Italy (40, 41) and also relevant globally (40). As a consequence, the guidelines do not recommend screening for thyroid cancer.

Alcohol is a well-recognized risk factor for esophageal and liver cancers. In Italy, between 1970 and 2014, the average alcohol consumption per capita (liters of ethanol per person per year) dropped from 14 to slightly over 6 L (42); the prevalence of drinking was significantly lower in women compared to men [2016 estimates *M* = 81% vs. *F* = 54% (43)]. Interestingly, the decline in alcohol consumption in both sexes correlated with the falling trend observed for esophageal and laryngeal malignancies (44, 45). It is therefore conceivable that the decline in the incidence rate recorded by RTV for esophageal and liver cancers is linked to the decrease in alcohol and tobacco use.

The decreasing trend observed for laryngeal cancer was almost twofold in males compared to females, whose higher smoking rates potentially reduced the benefits associated with lower exposure to tobacco genotoxicity. The observed decrease among women, which seems inconsistent with the reported increase in female smoking, could be explained by a reduction in the fraction of cases attributed to alcohol consumption (46). Alcohol was found to have a multiplicative effect on the risk of developing these cancers when combined with smoking (47–50). Hence, the decrease in alcohol consumption may have lessened this multiplicative effect.

Italy has one of the highest rates of liver cancer in the world, outside of Asia (51). In the addressed population, the burden of liver malignancy was considerably higher among males. The increasing incidence recorded in the decade 1987–1997, was followed by a continuing decline from the late 1990s. The lowering incidence rate was clearly more significant in males. Environmental infectious (i.e., viruses) and chemical/hepatotoxic factors (alcohol in particular) are the main etiological agents of cirrhosis and ultimately of its progression to cancer (i.e., hepatocarcinoma). Intrahepatic cholangiocarcinoma (by far less incident) and hepatocarcinoma are recorded together by RTV (i.e., C22). Routine screening in blood transfusions and the advent of the anti-hepatitis B virus (HBV) vaccination in 1980 (52) conceivably played a crucial role in reducing the risk of infectious cirrhosis (HBV, HCV), ultimately lowering liver cancer incidence. As for the etiological role of alcohol intake, Veneto has one of the three regional populations with the highest alcohol intake per capita in Italy (53). However, national data point to a decline in alcohol consumption (70% lower) from 1970 to 2010 (54), to which the declining trend in liver cancer incidence during the same time period could be attributed.

Since 2007, both sexes showed a decreasing incidence of colorectal (CRC) and anal malignancies, with a slightly earlier, more pronounced downward trend in males. In the early 2000s, the regional public health system established a free-access project for secondary prevention of CRC, consisting of a two-step procedure combining a fecal immunochemical test (FIT) with a second-step colonoscopy for FIT-positive patients. The reduction in CRC risk resulting from endoscopic removal of adenomas may explain the declining trend observed from 2007 onwards. Notably, the decrease in the incidence of CRC observed in the current study started earlier in males than females and was also more evident, apparently contrasting with higher compliance with screening among women in our region (55). Underpinning this unanticipated epidemiological evidence are several biological and clinical reasons, which should be addressed by cancer screening guidelines (56–61).

### 4.3 Increasing incidence trends in both sexes

Several cancer sites showed increasing incidence trends of varying magnitudes in both sexes. Cutaneous malignant (invasive) melanoma (CMM) showed the sharpest increase in the years considered in the study (1987–2019), with males experiencing the greatest annual change. The present findings are consistent with previous Italian and European studies, which reported a steadily increasing incidence in both sexes from 1990 to 2015 (62, 63). The more significant upward trend in males has been interpreted to stem from the potential removal of pre-invasive lesions associated with women’s attitude toward CMM prevention (use of sunscreen and “skin awareness”) and their higher propensity to perform skin self-examination (64–67). Recent evidence, however, supports the hypothesis that host-related biological factors (hormones, immune homeostasis, oxidative stress response, and X-linked genes) may play a sex-dependent “protective” role (68–70).

### 4.4 Sex-linked hormone-dependent cancers

Hormone-dependent malignancies contribute substantially to the overall incidence trends.

The incidence of breast cancer, accounting for more than one-third of female cancers in 2019, displayed a slight but steady increase from 1987 to 2019. Similar findings were observed in the UK (71), Germany, and other European countries (72) in the same period.

The different Local Health Units of the Veneto Region started breast cancer screening programs between 1999 and 2009. However, according to a survey by the National Institute of Statistics in 1999–2000, 67% of 50–69-year-old women referred that they had undergone at least one mammography in their life, in the absence of signs or symptoms (73). This is in line with the increase in incidence rates observed until 2000. The coverage of the regional female population with mammography after the spread population-based screening programs increased up to values higher than 80% (74).

Corpus uteri cancer, the fourth most common malignancy in women in 2019, showed constant growth over the whole-time interval considered. Similar trends were reported in other high-income countries, due mainly to the increasing incidence of endometrial cancer (75–77). This growing incidence is—at least in part—due to “epidemic” risk factors such as obesity and physical inactivity (78–83).

As in other European countries and worldwide (84), the incidence of prostate cancer rose significantly from 1987 to 2003, followed by a rapid decrease and a plateau, which remained unchanged throughout the 2010s. As seen in thyroid cancer, the marked change in prostate cancer incidence reported in the present study is conceivably the result of modifications in diagnostic criteria and clinical strategies for patient management. The increasing incidence resulting from the widespread spontaneous uptake of prostate-specific antigen (PSA) testing rapidly declined when the diagnostic reliability of PSA testing was clinically reconsidered (84–86).

### 4.5 All tumor sites

Consistently with long-term trends reported in Europe (5), the USA and Canada (87, 88), the present results show a decreasing trend in incidence for all cancer sites in males; the stable trends in females have consequently narrowed the sex gap in cancer incidence.

### 4.6 Limitations

Some limitations need to be considered when interpreting the findings of the present study. To start with, the present study focused only on cancer incidence trends in northern Italian region and may not be representative of other regions or on a global scale. However, its population-based design minimizes the risk of selection bias and the use of standardized algorithms reduced measurement variability, hence increasing the reliability of the values. Secondly, error in diagnosis is possible, but probability is very small given the high percentage of microscopically verified cases (88%) and the low percentage of death certificate only cases (<1%). At last, we fixed a significance level of 0.05 for each test, so some results might be statistically significant due to chance.

## 5 Conclusion

In the Veneto Region, the risk of lung cancer continues to increase in women, similar to other cancers associated to smoking, such as bladder and oral cancer. Greater efforts should be put in place to promote effective interventions to avoid and quit smoking, particularly in women. Prevention activities need to take into account sex differences in the psychological attitude that leads to smoking (for example, women are more likely to use tobacco to cope with negative feelings), in the propensity to quit smoking (e.g., women show more severe withdrawal symptoms and cravings to smoke, especially in relation to the luteal phase of the menstrual cycle), and in the level of smoking cessation (e.g., pregnancy promotes smoking cessation among women to a greater extent than becoming a parent does among men) (89).

Our data suggest the utmost importance of health promotion strategies for the prevention of melanoma in both sexes, aimed at spreading the knowledge on risk factors (i.e., ultraviolet (UV) radiation) and on protection strategies, including the UV index (90).

Finally, the growing trend of breast cancer suggests promoting both population-based educational interventions, such as increasing physical activity and reducing BMI and alcohol intake, and an approach with systematic targets or precision prevention(91).

The marked decline in the incidence of several cancer sites (e.g., esophagus, stomach, liver, larynx, oral cavity, lung in males, bladder, and leukemia) plausibly stems from the lessening impact of environmental risk factors. The secondary prevention strategy adopted for CRC has shown its well-recognized beneficial effect. In a minority of cancer sites (e.g., thyroid, prostate), the downturn in trend has been due mostly to changes in diagnostic criteria.

The findings of the present study contribute to understanding the background to different trends in cancer incidence by sex, and prompt the promotion of high-resolution cancer registration. Combining incidence and mortality rates with high-resolution patient profiles has the potential to crucially inform sex-tailored strategies for primary and secondary cancer prevention.

## Data availability statement

The datasets presented in this study can be found in online repositories. The names of the repository/repositories and accession number(s) can be found at: Figshare (https://figshare.com/s/2b061e3d3825a3556154).

## Ethics statement

Ethical approval was not required for the study involving humans in accordance with the local legislation and institutional requirements. Written informed consent to participate in this study was not required from the participants or the participants' legal guardians/next of kin in accordance with the national legislation and the institutional requirements.

## Author contributions

AB: Conceptualization, Methodology, Project administration, Writing – original draft, Writing – review & editing. GL: Conceptualization, Data curation, Formal analysis, Methodology, Visualization, Writing – original draft, Writing – review & editing. MZ: Data curation, Formal analysis, Writing – review & editing. EB: Data curation, Formal analysis, Writing – review & editing. SM: Conceptualization, Funding acquisition, Methodology, Writing – review & editing. CT: Conceptualization, Methodology, Visualization, Writing – review & editing. VB: Conceptualization, Methodology, Supervision, Writing – review & editing. SG: Data curation, Formal analysis, Writing – review & editing. MR: Data curation, Formal analysis, Supervision, Writing – review & editing.
