# Supplementary material for: Thirty-two-year trends of cancer incidence by sex and cancer site in the Veneto Region from 1987 to 2019
Source: Front Public Health. 2024 Jan 8;11:1267534. doi: 10.3389/fpubh.2023.1267534 (PMC10800396; doi:10.3389/fpubh.2023.1267534)
Supplement: Supplementary file 1 [file Data_Sheet_1.PDF]

Supplementary Material

Supplementary Table 1. Average population covered by the Veneto Cancer Registry (RTV)

**Years**	**Average population covered by RTV**	**% coverage**
1987	1,154,307	27%
1988-1989	1,455,439	33%
1990-1997	1,970,830	45%
1998-2007	2,239,770	49%
2008-2012	2,577,652	53%
2013	4,687,492	96%
2014-2019	4,892,209	100%

2 **Supplementary Table 2.** Age-standardized incidence rates (ASR) for the historical area (HA) and whole region for the main cancer sites. Period 2014-2019. Standardized ratio per 100,000 residents, European 2013 Standard Population

	Men				Women			
	**HA**	**Region**	**Rate ratio**	**p-value**	**HA**	**Region**	**Rate ratio**	**p-value**
All malignant tumours *excluding in-situ* cutaneous melanoma and non-melanoma skin cancer (C44)	690.0	690.7	1.001	0.8585	516.5	512.8	0.9929	0.2377
Breast (C50)					173.1	169.9	0.9816	0.0796
Prostate (C61)	148.3	147.4	0.9942	0.6407				
Colon, rectum and anus (C18-C21)	79.5	79.9	1.004	0.8182	50.8	50.1	0.986	0.456
Urinary Bladder (C67, D09.0, D41.4)	66.4	66.5	1.0021	0.9203	15.1	14.0	0.931	0.0433
Lung, bronchus and trachea (C33, C34)	83.0	84.0	1.0117	0.489	34.3	33.6	0.9798	0.3825
Invasive cutaneous melanoma (C43)	33.4	31.9	0.9564	0.0886	25.8	24.6	0.9535	0.0963

The rate ratios between the standardized rates in the historical area and the whole region showed no significant differences. For all malignant tumours, the standardized rate in the historical area was 690.0 vs 690.7 for the whole region in men, 516.5 vs 512.8 in women, with rate ratios of 1.001 and 0.9929, respectively (both p-values are higher than 0.05).
